# Enhanced Removal of Antibiotic in Wastewater Using Liquid Nitrogen-Treated Carbon Material: Material Properties and Removal Mechanisms

**DOI:** 10.3390/ijerph15122652

**Published:** 2018-11-26

**Authors:** Yaohui Wu, Wen Liu, Yonghong Wang, Xinjiang Hu, Zhengping He, Xiaoyong Chen, Yunlin Zhao

**Affiliations:** 1 College of Life Science and Technology, Central South University of Forestry and Technology, Changsha 410004, China; wyh752100@163.com (Y.W.); 15197704556@163.com (W.L.); bionano@163.com (Y.W.); xjhu@csuft.edu.cn (X.H.); 15673775866@163.com (Z.H.); 2 Hunan Research Center of Engineering Technology for Utilization of Environmental and Resources Plant, Central South University of Forestry and Technology, Changsha 410004, China; 3 College of Arts and Sciences, Governors State University, University Park, Illinois, IL 60484, USA

**Keywords:** liquid nitrogen, modified carbon, ampicillin, adsorption

## Abstract

Antibiotic residues in the aquatic environment have become a global problem posing a serious threat to the environment and an inherent health risk to human beings. In this study, experiments were carried to investigate the use of carbon material modified by liquid nitrogen treatment (CM1) and carbon material unmodified by liquid nitrogen treatment (CM2) as adsorbents for the removal of the antibiotic ampicillin from aqueous solutions. The properties of the CMs (CM1 and CM2) and the effects of variations of the key operating parameters on the removal process were examined, and kinetic, isothermal and thermodynamic experimental data were studied. The results showed that CM1 had larger specific surface area and pore size than CM2. The ampicillin adsorption was more effective on CM1 than that on CM2, and the maximum adsorption capacity of ampicillin onto CM1 and CM2 was 206.002 and 178.423 mg/g, respectively. The kinetic data revealed that the pesudo-second order model was more suitable for the fitting of the experimental kinetic data and the isothermal data indicated that the Langmuir model was successfully correlated with the data. The adsorption of ampicillin was a spontaneous reaction dominated by thermodynamics. In synthetic wastewater, CM1 and CM2 showed different removal rates for ampicillin: 92.31% and 86.56%, respectively. For an adsorption-based approach, carbon material obtained by the liquid nitrogen treatment method has a stronger adsorption capacity, faster adsorption, and was non-toxic, therefore, it could be a promising adsorbent, with promising prospects in environmental pollution remediation applications.

## 1. Introduction

In recent years, antibiotic residues in the aquatic environment have become a persistent, bioaccumulation problem, potentially toxic in the medium to long term. China has a large consumption of, estimated to account to 1/4 of the total amount of antibiotics consumed globally every year [1]. Unfortunately however, due to poor adsorption in the gut of the animals, elimination of the antibiotics occurs and the majority are excreted unchanged in feces and urine. In addition, some antibiotics are dropped directly into the water by fish farmers, leading to masses of antibiotics present as the unmodified parent compound [2]. In the past years, it was recognized that antibiotics represent a new source of water contaminants [3]. Antibiotics have been frequently detected in wastewater treatment plants (WWTPs) and in rivers [4]. β-Lactams, as a low cost class of antibiotics with broad-spectrum applicability are commonly used around the world [5], and have already caused some environmental problems. The occurrence of β-lactam antibiotic-resistant bacteria in surface water, animal waste water and poultry farms was already detected [6]. Moreover, β-lactam antibiotic-resistant bacteria are also found in the bodies of organisms, such as in humans [7] and fishes [8]. Considering the treat that residual β-lactam antibiotics pose to the environment and the risk to human beings [9], the development of efficient removal techniques is becoming a matter of significance to be addressed.

A number of methods were reported in the literature for the removal of β-lactam antibiotics from water. Arslan-Alaton and Caglayan demonstrated that ozone produced by an advanced oxidation method had positive effects on penicillin removal from formulation effluent [10]. Giraldo-Aguirre and collaborators evidenced that the β-lactam antibiotics could be removed efficiently from pharmaceutical industry wastewater by a photo-Fenton method at near neutral pH [11]. Biodegradation can be used as a common method to degrade β-lactam antibiotics, for example, degrading ampicillin in aqueous solutions using biofilms [12]. Limousy et al. efficiently eliminated amoxicillin from aqueous solutions with an adsorption method, and the elimination rate was shiwn to be as high as 93% [13]. Among all these methods, adsorption represents an ideal method due to its easy operation, high efficiency, and lack of formation of toxic byproducts [14]. Carbon is widely used as adsorbent, and its adsorption capacity could be contributed to two major factors: surface groups and specific surface area. Previous studies showed that the adsorptivity of carbon was closely related to its surface groups [15]. In order to increase the adsorption capacity, many methods have been used to produce various activated carbons by changing the surface groups of the carbon. Moussavi et al. [16] compared the adsorption of β-lactam antibiotics on NH_4_Cl treated activated carbon (NAC) and standard activated carbon (SAC). Over 99% of 50 mg/L β-lactam antibiotics were adsorbed using NAC at 0.4 g/L, while under similar experimental conditions only about 55% of β-lactam antibiotics was adsorbed by SAC. Although there have been some achievements in this area, many shortcomings still exist, such as the use of non-environmentally friendly denaturants (H_2_SO_4_, NH_4_Cl, (NH_4_)_2_S_2_O_8_), complex processes and high cost. Also it was well known that the adsorption properties of carbons depended heavily on their specific surface area. Due to its large surface specific area and uniform aperture, mesoporous carbon materials are promising adsorbents for the removal of antibiotics from aqueous solution [17]. Therefore, how to increase the specific surface area has become a research hotspot. Liang et al. [18] reported that the specific surface area of mesoporous carbon materials was not only related to the chemical composition of the raw precursor, but also to the preparation conditions. They increased the specific surface area by adjusting the ratio of water and ethanol, the amount of hydrochloric acid, and the carbonization temperature. However, in the high temperature sintering process, the carbon frameworks tended to shrink, which reduced the pore size and the surface area [19]. Recently, it was found that liquid nitrogen could be applied as passivator during the preparation of Ti-Al_2_O_3_ composites by forming a passivation layer (Ti-N) which protected the original structure [20], but a possible protective effect of liquid nitrogen on the carbon structure in the preparation of mesoporous carbon has yet to be reported.

The present study aimed at directly comparing adsorptive ampicillin removal with liquid nitrogen treated mesoporous carbon material and that obtained by a common method. The primary goals were to: (1) investigate the influence of liquid nitrogen on the carbon structure and (2) reveal the mechanism(s) of ampicillin removal by carbon materials.

## 2. Materials and Methods

### 2.1. Preparation and Characteristics of Carbon Materials (CMs)

Two types of CMs were synthesized by a soft template method [21]. The preparation process consisted of the following steps: Pluronic F127 and phloroglucin were successively added into a beaker with ethanol. Then HCl was gradually added to the mixture with vigorous stirring for 90 min at 25 °C. As the reaction proceeded, the homogeneous reactant mixture was separated into two layers. The upper clear fluid was poured out and the sediment was put in a crucible for drying 120 min at 60 °C in an oven (WGL-230B, TAISITE, Tianjin, China). The dried products were subsequently processed in two different ways. CM1 was obtained by placing the mixture in liquid nitrogen for half an hour and then calcining the mixture in a muffle furnace (SXL-1200M, SIOMM, Shanghai, China) at 800 °C for 3 h. CM2 was obtained by calcining the mixture in a muffle furnace at 800 °C for 3 h.

The specific surface area by the Brunauere-Emmette-Teller (BET) method, average pore diameter and total pore volume were detected by a surface and porosity analyzer (ASAP2020, Micromeritics Co., Shanghai, China). The morphology of the CMs was obtained by scanning electron microscopy (SEM, S-3400N, Hitachi, Tokyo, Japan). Changes of functional groups before and after ampicillin adsorption were determined by Fourier transform infrared spectroscopy (FT-IR, ALPHA, Beijing, China).

### 2.2. Detection of Ampicillin

The concentration of ampicillin in the aqueous phase was detected by a UV-spectrophotometry method (Nanodrop 2000/2000c, Thermo, Hong Kong, China). Ampicillin solution was mixed with imidazole solution [22] for 25 min at 60 °C, and then the UV-Vis absorption at 342 nm was recorded by a Nanodrop 2000 UV-Vis spectrophotometer (Nanodrop 2000/2000c, Thermo, Hong Kong, China). The absorption value corresponds to the concentration of ampicillin. Deionized-water produced by a pure water preparation system (FDY1001-UV-P, FLOM, Qingdao, China) was used in all experiments. The concentration of ampicillin in synthetic wastewater (SW) was detected at 220 nm using a High Performance Liquid Chromatography (HPLC) system (Agilent 1200, California, USA) [23]. A C_18_ column (5 µm particle size, 4.6 mmol/L × 250 mmol/L) was utilized. The mobile phase consisted of 0.02 mol/L potassium dihydrogen phosphate buffer and acetonitrile (92:8, *v*:*v*) delivered at a flow rate of 1.0 mL/min at 25 °C. It was filtered through a 0.45 µm size membrane filter (HDG-1A, Huadan, Beijing, China) and degassed before use.

### 2.3. Influence of pH on Adsorption of Ampicillin

The initial pH is regarded as the most important control parameter in the adsorption process. To determine the influence of pH on the adsorption of ampicillin, the pH in the experiments was adjusted to 2–9 using 1 mol/L NaOH or 1 mol/L HCl solution, using 1 mL solution of ampicillin with an initial concentration of 3 mmol/L and 5 mg adsorbent mixed in a 1.5 mL Eppendorf (EP) tube for 0.5 h at 25 °C.

### 2.4. Kinetic, Equilibrium and Thermodynamic Studies for Ampicillin Adsorption

To examine the adsorption of ampicillin on each carbon material, kinetic, equilibrium and thermodynamic studies were performed in batch experiments using 1.5 mL EP tubes. The experiments were conducted in triplicate at pH 7. Unless stated otherwise, after the adsorbent was completely mixed with the solution, the tubes were left standing still for a certain time before further use.

The kinetic study was performed to observe the effect of the reaction time on ampicillin adsorption. The influence of the adsorption time was confirmed by applying 1 mL solution of ampicillin with an initial concentration of 3 mmol/L and 5 mg adsorbent, mixed at 25 °C and pH 7 for 1–10 min. Samples were collected after the required reaction time. The results were analyzed by pseudo first-order and pseudo second-order kinetics.

The equilibrium study was conducted to investigate the influence of the equilibrium concentrations of ampicillin on the adsorption results. The experiments were performed at an equilibrium concentration of ampicillin of 0.5–3 mmol/L with carbon material doses of 5 mg in 1 mL of solution. The tubes were sampled after 0.5 h. The results were analyzed by the models of Langmuir and Freundlich.

The thermodynamic study was performed to examine the impact of temperature on the adsorption. The effect was determined by utilizing 1 mL solution of ampicillin with an initial concentration of 3 mmol/L and adsorbent amount of 5 mg in 1 mL solution mixing for 0.5 h at pH 7. A HH-2 digital electric-heated thermostatic water bath (HH-2, Guohua, Jiangsu, China) was used for controlling the reaction temperature.

### 2.5. Ampicillin Adsorption in SW

A typical SW was prepared according to the composition of the effluent from a sewage treatment plant in Hong Kong using glucose, ammonium chloride, urea, sodium nitrate and potassium dihydrogen phosphate [24]. The concentration of each compound was: 60 mg/L total organic carbon (TOC), 51 mg/L total nitrogen (TN), 5 mg/L total phosphorus (TP), and of these, the total nitrogen was composed of 40 mg/L ammonia nitrogen, 10 mg/L organic nitrogen and 1 mg/L nitrate nitrogen. In this experiment, 5 mg CM1 and 1 mmol/L ampicillin was added into SW and pure water, and then the effect of SW on the adsorption was studied.

### 2.6. Renew and Reuse of CMs

After the adsorption experiments, the used CMs were immersed in pure water for 30 s with vortex mixer, centrifuged (10,000 r/min, 5 min), and then collected. The desorption procedures were repeated four times before the CMs were gathered and dried at 60 °C. Thus, recovered CMs have been obtained. In the reuse of CMs experiments, the recovered CMs were applied to ampicillin solutions at the natural pH. The adsorption/desorption procedures were repeated ten times to evaluate the reusability of CMs.

## 3. Results

### 3.1. Characteristics of the Carbon Material

**Figure 1:**
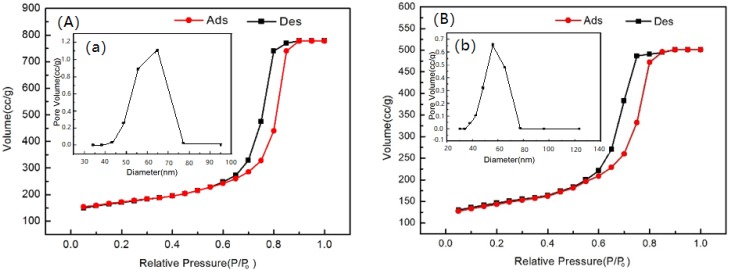
N_2_ adsorption-desorption of CMs: (**A**) CM1; (**B**) CM2. The inset graph shows pore size distribution of CMs: (**a**) CM1; (**b**) CM2.

The N_2_ adsorption-desorption isotherms of the tested CMs are provided in Figure 1, and the pore size distribution of the CMs is shown in the inset graph of Figure 1. Textural properties, e.g., specific surface area (S_BET_, m^2^/g), total pore volume (V_Total_, cm^3^/g), and micropore volume (V_Mes_, cm^3^/g) of the carbonaceous materials are listed in Table 1.

**Figure 2:**
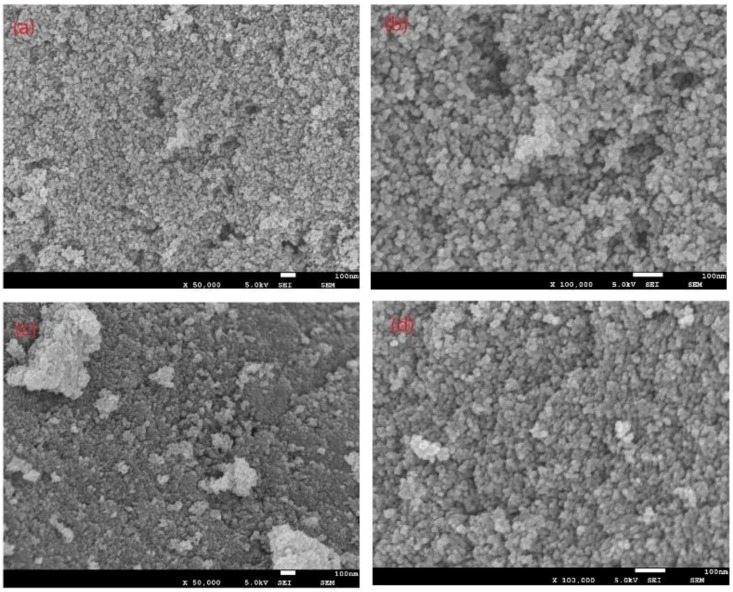
Analysis of CMs using SEM: (**a**,**b**) CM1; (**c**,**d**) CM2.

The porous structure, presented in Figure 1, revealed the nitrogen adsorption and desorption isotherms could be categorized as type IV isotherms. The nitrogen adsorption-desorption curve was related to the volume and size of the pores, so the textural properties of CMs could be known by analyzing the adsorption and desorption isotherms. According to the nitrogen adsorption curve and the nitrogen desorption curve, the nitrogen adsorption-desorption curve can be divided into five types: type I, type II, type III, type IV and type V [25]. The porous structure presented in Figure 1, revealed the nitrogen adsorption and desorption isotherms were categorized as type IV isotherm which indicates a mesoporous material [26]. As shown in Table 1, the effect of the liquid nitrogen treatments [27] on the textural properties of CM1 could also be observed. CM1 provided a higher surface area (550.52 m^2^/g) and total pore volume (1.20 cm^3^/g) than those provided by CM2 (S_BET_ = 422.93 m^2^/g and V_Total_ = 45.83 cm^3^/g), and the mean pore sizes of CM1 and CM2 were 62.98 nm and 45.83 nm, respectively. Figure 2 depicts two successive magnifications (×50 k and ×100 k) of the tested CMs. As shown, it was clear from the ×100 k magnification, that for CM1, the pore structure and the surface were looser and smoother compared with CM2, which exhibited a compact and irregular surface. Thus, CM1 with wider interstitial spaces displayed a larger surface area and bigger pore width. These results were in agreement with the previous N_2_ adsorption-desorption results of the CMs.

**Figure 3:**
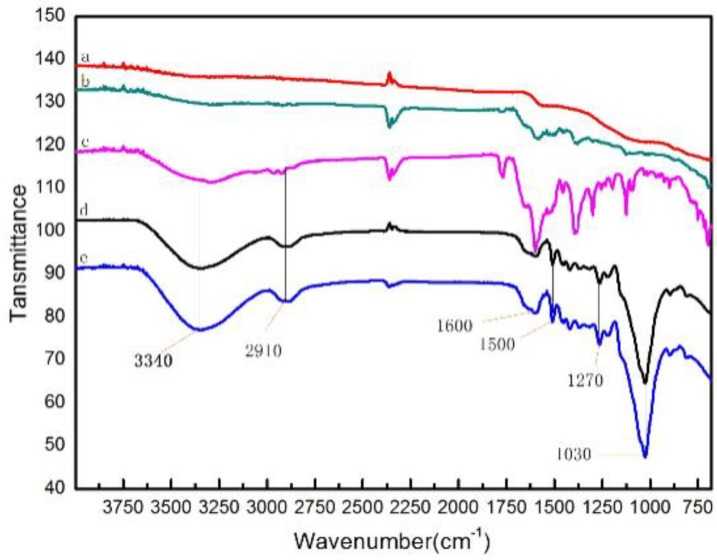
FTIR spectra of CM1 and CM2 before and after the adsorption of ampicillin: (a) CM2; (b) CM1. (c) ampicillin; (d) CM2 + ampicillin; (e) CM1 + ampicillin.

FTIR spectrum analysis helps determine the chemical compositions of materials. The FTIR spectra of the CMs before and after the adsorption of ampicillin in the wavelength range from 650 to 4000 cm^−1^ are shown in Figure 3.

As shown, the FTIR spectra of the CMs were the same. However, the functional groups of the CMs before and after the adsorption of ampicillin were rather distinct. After adsorption, there were new peaks at 1270 cm^−1^ which are characteristic of N-H stretching vibrations, suggesting the presence of the secondary amide. The absorption band at 1600 cm^−1^ in all the FTIR spectra and 1500 cm^−1^ was assigned to C-H stretching vibrations of the benzene ring. The medium peak at 2910 cm^−1^ attributed to C-H stretching vibrations, indicating the presence of alkane/alkene groups on the activated carbons surface. The absorption band at 3340 cm^−1^ is usually ascribed to -OH stretching vibrations of alcohol, carboxylate, ester and/or ether groups [28]. The small peak at 1030 cm^−1^, probably due to the infrared spectrum of C-S displacement toward low frequency by the effects of hydrogen bonds and ionic bonds.

### 3.2. Effect of pH

**Figure 4:**
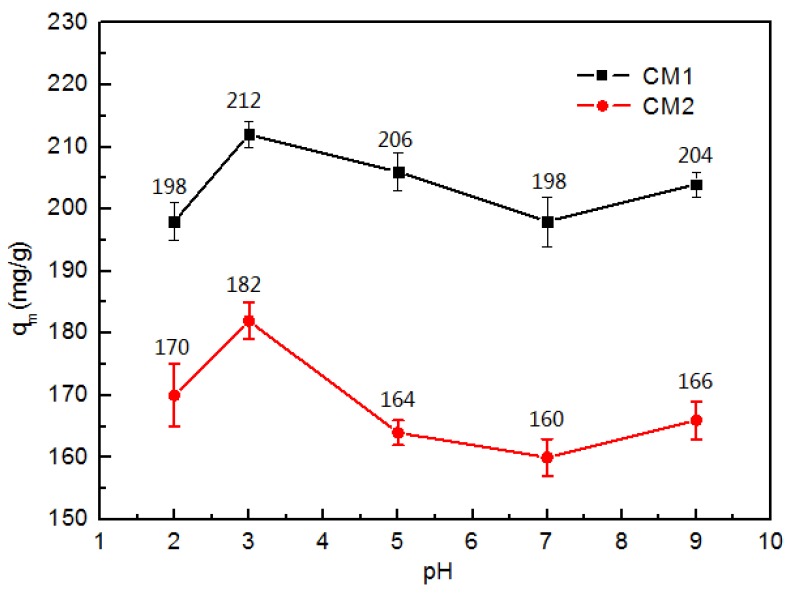
Effect of initial solution pH on the extent of adsorption of ampicillin (concentration of CMs = 5 g/L, concentration of ampicillin = 3 mmol/L, contact time = 30 min, at 25 °C).

The effect of initial solution pH on the extent of adsorption of ampicillin is shown in Figure 4. Within the pH range studied (pH 2–9), both CMs reached the maximum adsorption when the pH was 3. Ampicillin (pKa = 2.96 for its free carboxylic acid groups and pKa = 7.22 for its free amino groups) is ionized in solution. At pHs between 2.96 and 7.22, ampicillin presents a zwitterionic structure [29]. As the pH increases, the structure of ampicillin could be strongly affected by the pH of the solution and the activated carbon become negatively charged.

### 3.3. Ampicillin Adsorption: Kinetic, Isotherm and Thermodynamic Analysis

The adsorption kinetics of ampicillin on the carbon materials was evaluated. Both 1st order and 2nd order adsorption kinetics were determined using Equations (1) and (2), respectively.

The pseudo-first-order expression is:

(1) $$\;{q_{t}=q_{e}\;(1\;-\;e}^{-k_{1}t})\;$$

where *q*_t_ (mg/g) is the concentration of ampicillin adsorbed mass per unit of the adsorbent at time *t* (min). *k*_1_ (1/min) is the pseudo-first-order adsorption rate constant, *q*_e_ (mg/g) is the adsorption capacity counted by the pseudo-first-order model.

The pseudo-second-order equation is as follows:

(2) $$\;q_{t}\;=\;\frac{\;q_{e}^{2}k_{2}t\;}{1+q_{e}k_{2}t}\;$$

where *q*_t_ (mg/g) is the concentration of ampicillin adsorbed mass per unit of the adsorbent at time *t* (min). *k*_2_ (g/mg/min) is the pseudo-second-order adsorption rate constant, and *q*_e_ (mg/g) is the adsorption capacity computed by the pseudo-second-order model.

**Figure 5:**
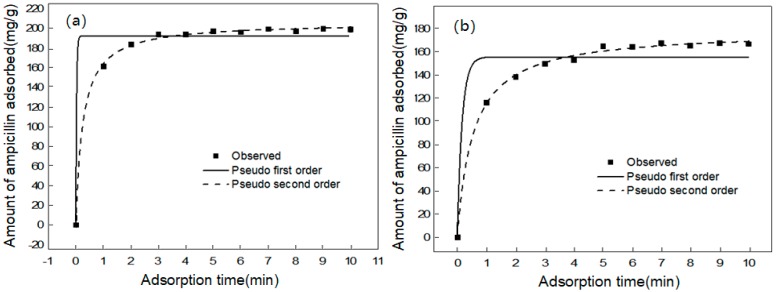
Kinetic modeling of ampicillin adsorption onto CMs: (**a**) CM1; (**b**) CM2. (concentration of CMs = 5 g/L, concentration of ampicillin = 3 mmol/L, pH = 7, at 25 °C).

The parameters found for the different models are presented in Table 2, Some kinetic curve fitting results are shown in Figure 5.

The influence of contact time on the adsorption of ampicillin by the tested CMs is depicted in Figure 5a,b for CM1 and CM2, respectively. First, an exponential increase in the adsorption was registered within the first 3 min for CM1 and within 5 min for CM2, and the maximum adsorption capacities of ampicillin on CM1 and CM2 was 206.002 mg/g and 178.423 mg/g, respectively. Such a tendency confirms the significant effect of the CMs’ properties (surface area and interstitial porosity) on the removal efficiency. These further proved that CM1 has a larger specific surface area and larger apertures than CM2, which could enhance the adsorption capacity of carbon materials and accelerate the adhesion of ampicillin to the carbon material [30]. The same result was previously reported in Table 1 regarding the surface area and pore volume. According to the values of *R*^2^ (Table 2), it was easily obtained that the pseudo-second order model was more appropriate for depicting both CMs’ kinetic data.

**Figure 6:**
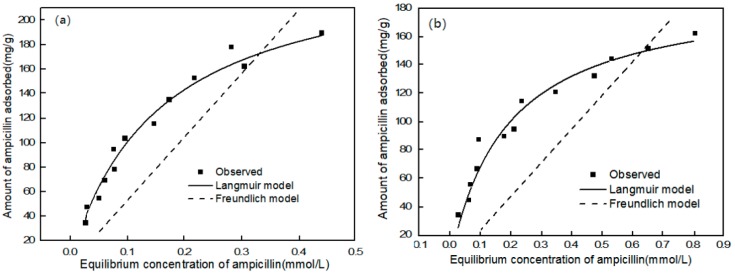
Equilibrium isotherm model of ampicillin adsorption onto CMs: (**a**) CM1; (**b**) CM2. (concentration of CMs = 5 g/L, contact time = 30 min, pH = 7, at 25 °C).

The Langmuir model (Equation (3)), and Freundlich model (Equation (4)) were used to further investigate the adsorption process and mechanism, with the results shown in Figure 6 and Table 3.

Langmuir isotherm:

(3) $$\;q_{e}\;=\;\frac{q_{L}k_{L}C_{e}}{1\;+\;k_{L}C_{e}}\;$$

where *q*_e_ (mg/g) is the ampicillin adsorption capacity at equilibrium, *q*_L_ (mg/g) is the maximum ampicillin adsorption capacity of CMs, and *k*_L_ (L/mg) is the constant that related to the rate of adsorption.

Freundlich isotherm:

(4) $$\;q_{e}\;=\;K_{F}C_{e}^{1/n}\;$$

where *q*_e_ (mg/g) is the quantity of ampicillin adsorbed on CMs, *C*_e_ (mg/L) is the equilibrium concentration of ampicillin, *K*_f_ and n are the Freundlich constants which are correspond to adsorption capacity and intensity, respectively.

As shown in Table 3, both *C*_e_/*q*_e_ versus *C*_e_ curve and *R*^2^ values indicate that the Langmuir isotherm model had a better fit than the Freundlich model. From the Langmuir model, the maximum adsorption capacity (*q*_m_) of ampicillin was calculated to be 252.819 mg/g for CM1, which was higher than that for CM2 (193.274 mg/g). This could be explained by the fact that CM1 had a larger specific surface area with more surface active sites after liquid nitrogen treatment. The mechanisms involved in the process of ampicillin adsorption and the adsorption capacity of CMs were determined by obtaining the corresponding adsorption isotherms. Figure 6 shows that the adsorption isotherms of ampicillin on the CMs could be classified as L-2 type, according to the Giles classification [31], suggesting that ampicillin molecules are adsorbed in parallel to the carbon surface and there is no major competition between ampicillin and water molecules for the active sites on the carbon.

Thermodynamic parameters, such as entropy change (△S°), enthalpy change (△H°) and Gibbs free energy change (△G°) were computed with Equation (5):

(5) $$\;\ln\;k_{d}\;=\;\frac{\;\Delta S{}^{\circ}\;}{R}\;-\;\;\frac{\;\Delta H{}^{\circ}\;}{\mathrm{RT}}\;$$

where *K*_d_ is the adsorption distribution constant (L/mg), R is the universal gas constant (8.314 J/mol K), and T is the absolute temperature (K). The adsorption distribution constant *K*_d_ is computed using the following Equation (6):

(6) $$\;K_{d}\;=\;\frac{\;C_{e,\mathrm{adsorption}\;}}{C_{e,\mathrm{solution}}}\;$$

where *C*_e_,_adsorption_ and *C*_e_,_solution_ are the equilibrium concentrations of ampicillin (mg/L) on the adsorbent (CMs) and in the solution, respectively.

The thermodynamic analysis is presented in Table 4. The maximum adsorption capacity was obtained at 55 °C, and the adsorption of ampicillin on CMs was affected as the temperature varied between the ranges of 35–55 °C. The negative values of Gibbs free energy change (△G°) (−5.812–−3.392 kJ/mol) at all temperatures indicated that ampicillin adsorption on CMs was a spontaneous reaction. CM1 and CM2 had a positive value of entropy (△S°), which indicated that the entropy increased at the solid-solution interface. The distribution of enthalpy (△H°) indicated that the adsorption process was endothermic and was more favorable at high temperatures, and might be caused by the changes in hydrogen bonding, protonation or Van der Waals forces.

### 3.4. Ampicillin Adsorption in SW

**Figure 7:**
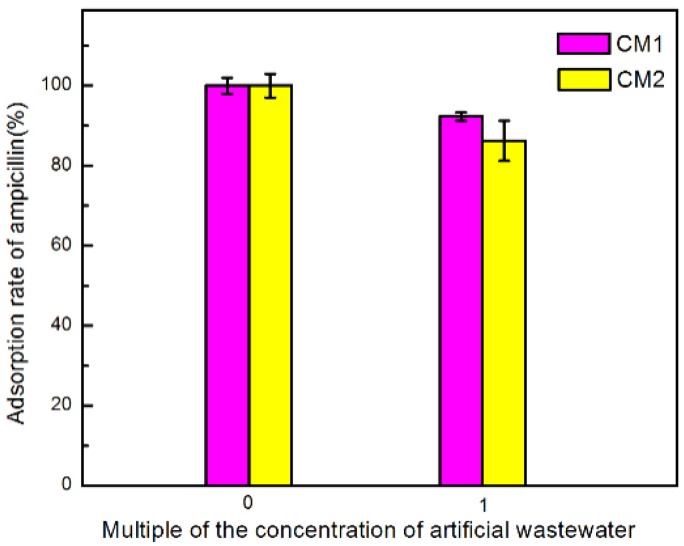
Removal rate of ampicillin in synthetic wastewater (concentration of CMs = 5 g/L, contact time = 30 min, at 25 °C).

In order to assess the feasibility of the CMs’ practical application, the CMs were used to remove ampicillin from SW to study the ampicillin removal rate. As shown in Figure 7, in SW, both CM could not completely remove the ampicillin, and the removal ratio of 1 mmol/L ampicillin was 92.31% for CM1 and 86.56 for CM2, respectively.

### 3.5. Renew and Reuse of CMs

**Figure 8:**
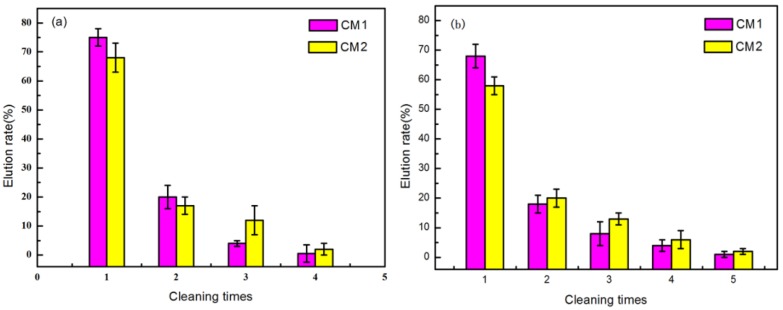
(**a**) Ampicillin elution from CMs in water; (**b**) Ampicillin elution from CMs in synthetic wastewater.

The experimental results of adsorption material regeneration were presented in Figure 8. Figure 8a shows the regeneration of CMs in pure water, and Figure 8b demonstrates the regeneration of the CMs in SW. As shown in Figure 8, the CMs could basically be regenerated after being washed only three times in pure water, however, CM2 needed four washes to reach the same goal in SW. This indicated that SW changed the interaction between sorbent and ampicillin, and decreased the effect of regeneration. The regeneration of CM1 under these two conditions were better than that of CM2.

**Figure 9:**
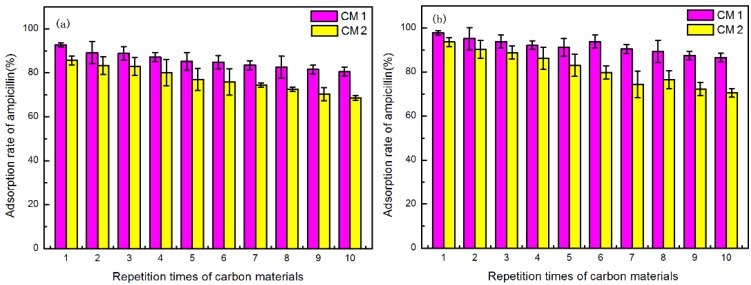
(**a**) Reuse of CMs in SW solution; (**b**) Reuse of CMs in normal SW (concentration of CMs = 5 g/L, contact time = 30 min, at 25 °C).

The reuse results of the CMs are shown in Figure 9, which indicates that after ten adsorption/desorption recycles, the ampicillin removal ratio with CM1 was higher than that with CM2. CM1’s removal ratio was above 80.56%, and that of CM2’s was nearly 68.57%. The good regeneration ability of CM1 made it suitable to capture ampicillin in sewage treatment, indicating that the modified method established in this work could be useful for practical application.

## 4. Discussion

Antibiotics are a global concern as emerging contaminants. In this study, ampicillin was removed from aqueous solutions using a novel carbon material. The textural properties of the carbon materials were characterized by N_2_ adsorption-desorption isotherms, SEM and FTIR and the ampicillin removal mechanism was explored by kinetic, equilibrium and thermodynamic studies. The carbon materials were further used to remove ampicillin from SW.

Phenolic resin is widely used for the preparation of carbon materials due to the fact it is rich in hydroxyl groups and could form hydrogen bonds with the PEO segment of the triblock copolymer, which was a driving force for forming a carbon material structure. In previous studies, the main method for improving carbon materials was to change the catalyst during the preparation process [32,33]. However, no similar reports have been reported to prevent sintering during the carbonization process. In the process of synthesizing Ti-Al_2_O_3_, Liu et al. used liquid nitrogen passivated Ti nanopowder to prevent sintering, and to prepare a material with uniform phase dispersion [20].

In this research, a new absorbent (CM1) had been obtained by modified liquid nitrogen treatment, which was utilized to remove ampicillin from solution in comparison with CM2 (obtained without liquid nitrogen treatment). Combined with N_2_ adsorption-desorption isotherms and SEM results, it was clear obtained that CM1 has a larger specific area and bigger pores than CM2. We confirm that liquid nitrogen could obviously increase specific surface area and aperture of CMs by forming a passivation layer as mentioned before [19]. According to the results of kinetic, equilibrium and thermodynamic studies, CM1 removed ampicillin more efficiently. Our results were consistent with others’ research, as Álvarez has indicated that the surface area was increased from 352 to 391 m^2^ g^−1^ by increasing the carbonization temperature of the carbon material, and the average pore width was changed from 0.86 nm to 1.31 nm. which led to an increase in the amount of tetracycline adsorbed from 53.9 to 58.1 mg g^−1^ [34].

Regarding the effect of contact time, concentration and temperature, the results for the case of ampicillin demonstrated that the kinetic data were successfully correlated with the pseudo-second order model and the isothermal data were well-fitted to the Langmuir isotherm model. The high accuracy in the prediction of experimental data by the pseudo-second order model has been considered in many research works [35] as evidence that chemical bonds are involved in the adsorption mechanism of ampicillin onto CMs. This assumption has been confirmed by the result of the study of the FTIR spectra of the adsorbed phase onto CMs (Figure 3), which displayed new chemical bonds between both the adsorbed phase and the adsorbent. The Langmuir isotherm model results was in agreement with previous studies [36], which indicated that adsorption was a monolayer adsorption and CMs have homogeneous solid surfaces.

The experimental pH data also revealed a feeble change between pH 2 and 9 and the adsorption reached a maximum at pH 3, indicating that the structure of ampicillin had a major effect on the adsorption of ampicillin on CMs [28].

According to our desorption and the renew-recycle experiments, CM1 could basically be regenerated in water after being washed only three times, however, with synthetic wastewater the adsoption by CM1 was still up to 86.56%, but down to 68.57% with CM2. Ncibi et al. have reported that carbon materials with larger pore sizes were easier to reuse as it was difficult to wash substances out from small sized pores [30]. According to a previous study [37], other substances present in the synthetic wastewater can compete for the sorption sites with ampicillin, so the removal ratio was decreased.

These experiments have some limitations. Phenolic resin is relatively expensive compared to other carbon raw materials (bioresidue and coal based), so the liquid nitrogen modification method should be further applied to other activated carbons forms.

The liquid nitrogen treatment method is a new green and low cost physical modification method. Compared with other chemical modification methods, it could produce carbon materials with larger specific surface area and more adsorption sites to increase the adsorption capacity without changing the chemical properties. If combined with other modification methods, a new modified material with better adsorption ability would be achieved. Moreover, the large surface area of carbon materials could improve the properties of batteries [38] and increase hydrogen storage capacity [39], so the liquid nitrogen treatment method for carbon material modification may have broader application prospect in sewage treatment and industrial manufacture.

## 5. Conclusions

In this research, liquid nitrogen was used as a passivating agent to modify carbon materials, and a new absorbent (CM1) was obtained, which was utilized to remove ampicillin from aqueous solutions. Compared with CM2, the specific area and aperture of CM1 was larger, which can remove ampicillin more efficiently. Kinetic data were successfully correlated to the pseudo-second order model. Moreover, the thermodynamic results were consistent with the Langmuir model. According to the model, the adsorption capacities of CM1 and CM2 were 212 mg/g and 182 mg/g, respectively. The whole adsorption process was an endothermic reaction. The adsorption capacity was affected by the solution pH, and it reached a maximum at pH 3. The ampicillin removal ratio of CM1 was higher than that of CM2, however, it decreased with increasing concentration of SW. In the renew-recycle experiments, the removal ratio of ampicillin by CM2 was still up to 80.56%, but down to 68.57% with CM1.

## Figures and Tables

**Table 1:** Characteristics of synthesized (CM1, CM2) mesoporous carbons.

Characteristic	CM1	CM2
BET surface area (m^2^/g)	550.52	422.93
Average pore width (nm)	62.98	45.83
Total pore volume (cm^3^/g)	1.20	0.84
Mesopore volume (Vp) (cm^3^/g)	0.84	0.71
Macropore volume (cm^3^/g)	0.36	0.13

**Table 2:** Kinetic parameters for the adsorption of ampicillin on CMs (concentration of CMs = 5 g/L, concentration of ampicillin = 3 mmol/L, pH = 7, at 25 °C).

CMs	the Pseudo-First-Order			the Pseudo-Second-Order		
	*k*_1_ (1/min)	*q* (mg/g)	*R* ^2^	*k*_2_ (g/mg/min)	*q* (mg/g)	*R* ^2^
CM1	36.632	192.257	0.961	0.019	206.002	0.999
CM2	6.036	155.728	0.885	0.010	178.423	0.998

**Table 3:** Adsorption isotherm parameters for the adsorption of ampicillin on CMs (concentration of CMs = 5 g/L, contact time = 30 min, pH = 7, at 25 °C).

CMs	Langmuir			Freundlich		
	*K*_L_ (L/mg)	*q*_max_ (mg/g)	*R* ^2^	*K*_F_(L/mg)	*n*	*R* ^2^
CM1	6.475	252.819	0.992	1.962	0.004	0.388
CM2	5.428	193.274	0.988	1.884	0.007	0.387

**Table 4:** Thermodynamic parameters for ampicillin adsorption on CMs (concentration of CMs = 5 g/L, concentration of ampicillin = 3 mmol/L, contact time = 30 min, pH = 7).

CMs	Temp. (°C)	△H (KJ/mol)	△S (KJ/mol)	△G (KJ/mol)
CM1	35	12.471	49.884	−3.392
	45	12.471	49.884	−3.89
	55	12.471	49.884	−4.612
CM2	35	8.314	43.232	−5.001
	45	8.314	43.232	−5.434
	55	8.314	43.232	−5.812
